# Distribution and Determinants of Cytomegalovirus Induced End Organ Disease/s among People Living with HIV/AIDS in a Poor Resource Setting: Observation from India

**DOI:** 10.1371/journal.pone.0117466

**Published:** 2015-02-13

**Authors:** Avirup Chakraborty, Tanmay Mahapatra, Sanchita Mahapatra, Sabbir Ansari, Sattik Siddhanta, Siwalik Banerjee, Dipanjan Banerjee, Rathindra Nath Sarkar, Subhashish Kamal Guha, Nilanjan Chakraborty

**Affiliations:** 1 ICMR Virus Unit, Kolkata, ID & BG Hospital, GB4, 57 Dr. SC Banerjee Road Beliaghata, Kolkata 700 010, India; 2 Jonathan and Karin Fielding School of Public Health, University of California Los Angeles, Los Angeles, California, United States of America; 3 Calcutta Medical College and Hospital, Department of general Medicine, Kolkata 700073, India; 4 Calcutta Medical College and Hospital, Apex Clinic, Kolkata 700073, India; 5 School of Tropical Medicine, Department of Virology, Kolkata 700 073, India; University of Regensburg, GERMANY

## Abstract

**Background:**

In India, despite well-established anti-retroviral treatment programs, Cytomegalovirus (CMV) infection-related end-organ diseases (EODs) still remain a major concern resulting in exacerbation of morbidity and mortality among HIV/AIDS patients. A prospective study was designed to understand the distribution and prognosis of CMV associated EODs and to determine a standardized cut-off value for serum CMV viral load associated with the development of EODs amongst HIV/AIDS subjects.

**Methods:**

In a cohort of 400 late-diagnosed HAART naïve HIV/AIDS subjects attending anti-retroviral centers of Kolkata during 2008–2014, the median duration of follow-up was 560 days, and at least 3 visits subsequent to the baseline were mandatory for eligibility. HIV-1 and CMV viral load were estimated by performing Real-Time Polymerase Chain Reactions (PCR).

**Results:**

Among subjects, 40.5% (162/400) had CMV EODs which were more common at lower CD4 counts. Poor prognosis and higher death rate were associated with a low CD4 count and increased HIV-1 and CMV viral loads. Subjects having higher CD4 count responded better to therapy [for CD4 = 60–100: Risk Ratio:RR = 1.48 (95% Confidence Interval: 95%CI = 1.18–1.82) and for CD4 = 30–59: RR = 1.64 (95%CI = 1.18–2.27)]. The cut off value of the serum CMV viral load (expressed as log10DNA/ml serum) associated with the development of EODs and disseminated CMV EODs was determined as 5.4 (p<0.0001) and 6.4 (p<0.0001) respectively. These cut offs were found to have satisfactorily high sensitivity, specificity, positive and negative predictive values.

**Conclusion:**

Prognosis of CMV EOD was poor as indicated by higher death rates among subjects with lower CD4 count, and specific cut-off values were found to have useful potential for identification and treatment of CMV infected HIV/AIDS patients in due time to avoid CMV EODs among HIV/AIDS subjects. Targeted intervention programs seemed to be required urgently to make these cut-offs operational in order to minimize the burden of CMV EOD in this vulnerable population.

## Introduction

Among HIV/AIDS patients, Cytomegalovirus (CMV) infection results in considerable exacerbation of morbidity and mortality associated with a wide range of clinical manifestations while disseminated CMV infection remains quite common [1]. The spectrum of these clinical manifestations of CMV infection individually or in combination with HIV/other opportunistic infections is collectively known as CMV-associated end-organ disease (EOD). The common components of these complicated clinical scenarios include retinitis, colitis, encephalitis, polyradiculopathy, esophagitis, pneumonia etc., while adrenitis and oral ulcers are encountered less frequently [2–10]. Study by Wohl et al (2005) revealed that detectable CMV-DNA in the plasma by polymerase chain reaction (PCR) could independently predict death amongst the AIDS subjects [11]. Different studies have also associated CMV as a cofactor for rapid HIV-1 disease progression [12–14]. Moreover, several studies by Crum et al (2006), Jain et al (2003) and Goldberg et al (2005) have reported a significant decrease in the incidence of CMV-related EODs and annual death rates amongst the HIV-infected patients after the availability of Highly Active Anti-Retroviral Therapy (HAART) compared to those in the pre-HAART era [15–17]. Subjects receiving HAART undergo a partial immune recovery as is manifested by a rise in the CD4+ T-lymphocytes count and a decrease in the serum HIV loads [18,19]. These studies have thus concluded that on adherence to HAART and with proper health care approach, decrease in CMV-associated complications is often noted.

In our study population cohort, comprising of 400 late diagnosed [mean baseline CD4+ cell count being 45 (range 4–100) at the time of their HIV-1 diagnosis and recruitment for the study] HAART naïve HIV/AIDS patients, 40.5% (162/400) subjects were diagnosed with different stages of CMV disease. This finding supported the fact that even with the advent of well-established treatment procedures, due to the persisting operational problems like late HIV/AIDS diagnosis, inaccessibility to proper health care, CMV-related EOD(s) still remained a major public health concern amongst the HIV/AIDS patients [20]. The magnitude of this problem thus calls for an effort to develop an insight into the CMV load threshold, beyond which CMV-associated EODs might be considered inevitable.

A prospective study was thus designed with the objective of defining a standardized serum CMV load cut-off value by real-time PCR that can be used as a threshold associated with the development of CMV-induced EOD and also the development of disseminated CMV-related EODs amongst the HIV/AIDS subjects. An idea of the serum cut-off value of CMV load may help the policy makers to undertake proper intervention strategy in due course of time so that surveillance and monitoring plan may be designed to keep the serum CMV load of HIV/AIDS patients below the identified cut-off value.

## Materials and Methods

### Ethics statement

Details of the study were explained to the subjects in a language that they understand completely and voluntary written informed consents were obtained from each and every subject maintaining confidentiality as per the standard national guidelines. The study content and procedures were approved by the Institutional Ethics Committees of the Indian Council of Medical Research (ICMR) Virus Unit, Kolkata, Calcutta Medical College, Kolkata and School of Tropical Medicine, Kolkata.

### Study subjects and eligibility

This study included 400 HAART naïve newly diagnosed HIV/AIDS patients having CD4 count 100 or below (Table 1), admitted to or visiting the Apex Clinic (outpatient department) of Medical College and Hospital and the ART Center in the School of Tropical Medicine, two referral centers in Kolkata for patients of HIV infection or AIDS, between July 2008 and June 2014. The median duration of study (follow-up) was 560 days (range 550–600 days) and each subject had a median of 6 visits during this study period, with a mean interval of 127 days between the visits. At least 3 study visits subsequent to the baseline visit were considered mandatory for being included in the analyses. No subject was lost to follow-up during this study.

**Table 1:** Distribution of the baseline characteristics of the participating HIV/AIDS patients (N = 400).

**Continuous Variables**	**Mean**	**Range**
Mean age in years	37	19–63
Mean baseline log10 HIV-1 RNA/ml serum	4.6	3.8–5.9
Mean baseline log10 CMV* DNA/ml serum	5.3	3.5–6.9
Mean baseline CD4+ cell count in cells/μl	45	4.0–100.0

**Categorical Variables**	**Categories**	**Frequency**	**Percentage**
Gender	**Male**	207	51.8
	**Female**	193	48.2
CD4+ Count at the time of diagnosis of HIV	**<30**	98	24.5
	**30–59**	210	52.5
	**60–100**	92	23.0
HIV viral load	**Detectable**	400	100.0
	**Non-detectable**	0	0.0
Subjects with detectable CMV* viral load	**Detectable**	337	84.3
	**Non-detectable**	63	15.7
Subjects with CMV* End organ diseases (EODs)	**Yes**	162	40.5
	**No**	238	59.5

*CMV: Cytomegalovirus

The baseline characteristics of the study subjects are listed in Table 1. HIV/AIDS patients without detectable CMV infection (63), with detectable CMV infection, but without any accompanying clinical manifestations (175) and who were diagnosed to have developed earlier stages of CMV disease (162) were included in the study. Subjects who presented with advanced CMV disease manifestations were excluded from the study.

### Measurement of whole blood CD4+ Lymphocyte count, HIV-1, CMV viral load

During the hospital visit, 3 ml of venous whole blood samples were collected and anti-coagulated with EDTA, from the eligible HIV-1 seropositive patients, as a part of the routine clinical care and were subjected to CD4+ count (expressed as cells/μl of blood) estimation procedure using FACS Calibur flow cytometer (Becton Dickinson, San Jose, California, USA). HIV-1 (determined as HIV-1 RNA/ml serum and expressed as log10 HIV-1 RNA/ml serum) and CMV viral load (determined as CMV DNA/ml serum and expressed as log10 CMV DNA/ml serum) were estimated by performing HIV-1 Real-Time PCR using Roche Diagnostics, Basel, Switzerland and CMV Real-Time PCR using Shanghai ZJ Biotech Company Ltd., Shanghai, China respectively in ABI Prism 7000 Real-Time PCR instrument, USA. Clinical diagnoses of CMV EODs and disseminated CMV EODs were used as the gold standard for determining the sensitivity and specificity of CMV viral load cut-off values to determine both the diseases respectively.

Threshold cycle number (CT) of triplicate reactions, was obtained and the mean CT of triplicate reactions was determined. From the standard curve, the HIV-1 and CMV loads were determined during each study visit.

### Statistical analysis

Descriptive analysis was done to determine mean (with range) values for continuous variables and proportions for categorical variables. The relation between mortality rates amongst subjects with CMV EOD/s and CD4 count was examined by using Kaplan–Meier method.

Statistical method used for the determination of cut-off value of serum CMV viral load for the development of CMV EOD/s was Receiver Operating Characteristic (ROC) curve.

The statistical analysis was done by using GraphPad Prism 5 and MedCalc softwares. Measures of association were reported as risk ratios (RR) and odds ratios (OR) with corresponding 95% confidence intervals (95%CI). ORs were determined in addition to RRs to make sure that any difference in measure due to inadequacy in sampling (denominator problem) or follow up may be compared. While all corresponding p values were reported, a p value < 0.05 was regarded as statistically significant.

Regarding prognosis of HIV, patients responded to therapy were defined as those who were found to have increased CD4 count compared to their baseline value whereas deterioration was defined as the reverse.

## Results

### Cytomegalovirus EOD/s

None of the 15.8% (63/400) subjects with undetectable CMV DNA in serum developed CMV EOD/s. 48.0% (162/337) of the subjects with detectable CMV DNA in the serum were diagnosed with CMV EOD/s. 35.8% (58/162) of these subjects presented with cases of isolated retinitis. Whereas, cases of CMV induced isolated Gastro intestinal tract (GIT) and Pulmonary diseases were present in 11.7% (19/162) and 6.2% (10/162) of these subjects respectively. Retinitis associated with other CMV EOD/s was also common. Retinitis associated with CMV induced GIT and Pulmonary diseases occurred in 25.9% (42/162) and 16.7% (27/162) of the subjects respectively. 3.7% (6/162) of the cases presented with a multisystem involvement of CMV induced Retinitis, GIT and Pulmonary diseases. 83.3% (5/6) of these subjects had CD4 <30 cells/μl and 16. 7% (1/6) had CD4 in the range between 30–59 cells/μl. Multisystem involvement (involving at least 2 systems) of CMV EODs was found in 67.6% (50 of 74) amongst the subjects having CD4<30 cells/μl who had developed CMV EOD/s—37.8% (28/74) had CMV induced GIT diseases along with retinitis while 22.9% (17/74) had developed pulmonary diseases along with retinitis. 6.8% (5/74) had CMV induced Retinitis, GIT and Pulmonary diseases. Rest, 32.4% (24/74) of the subjects had single system involvement, 20.3% (15/74) had developed isolated retinitis, 8.1% (6/74) isolated GIT and 4.1% (3/74) isolated pulmonary diseases. 35.7% (25/70) of the subjects with CMV EOD/s in the CD4 range from 30–59 cells/μl had multisystem involvements of CMV diseases- 20.0% (14/70) had CMV induced GIT diseases along with retinitis, 14.3% (10/70) had CMV induced pulmonary diseases along with retinitis and 1.4% (1/70) had developed CMV induced Retinitis, GIT and Pulmonary diseases. The rest 64.3% (45 of 70) of subjects, presented with a single system involvement of CMV EOD which included-45.7% (32/70) isolated retinitis, 11.4% (8/70) isolated GIT and 7.1% (5/70) isolated pulmonary diseases.

All (100%) of the 18 subjects with CD4 between 60–100 cells/μl presented with a single system involvement of CMV EOD which included 61.1% (11/18) isolated retinitis, 27.8% (5/18) isolated GIT and 11.1% (2/18) cases with isolated pulmonary disease. (Table 2)

**Table 2:** Distribution and Outcome of CMV End Organ Diseases across the strata of CD4 count among the participating HIV/AIDS patients having CMV EODs (N = 162).

Group	CD4 count	Total No. of patients	%	Organ involvement	No. of patients	Responded to therapy		Static		Deteriorating		Death	
						No	%	N	%	N	%	N	%
**A**	<30	74	45.7	Isolated Retinitis	15	6	40.00	2	13.33	2	13.33	5	33.33
				Isolated GIT	6	3	50.00	-	-	1	16.67	2	33.33
				Isolated pulmonary	3	1	33.33	-	-	1	33.33	1	33.33
				Retinitis and GIT	28	14	50.00	4	14.29	6	21.43	4	14.29
				Retinitis and pulmonary	17	6	35.29	3	17.65	6	35.29	2	11.76
				Retinitis, GIT and pulmonary	5	-	-	-	-	2	40.00	3	60.00
				Total	74	30	40.54	9	12.16	18	24.32	17	22.97
**B**	30–59	70	43.2	Isolated Retinitis	32	23	71.88	4	12.50	4	12.50	1	3.13
				Isolated GIT	8	5	62.50	1	12.50	1	12.50	1	12.50
				Isolated pulmonary	5	3	60.00	-	-	1	20.00	1	20.00
				Retinitis and GIT	14	11	78.57	1	7.14	1	7.14	1	7.14
				Retinitis and pulmonary	10	4	40.00	2	20.00	2	20.00	2	20.00
				Retinitis, GIT and pulmonary	1	-	-	-	-	-	-	1	100.00
				Total	70	46	65.71	8	11.43	9	12.86	7	10.00
**C**	60–100	18	11.1	Isolated Retinitis	11	9	81.82	-	-	2	18.18	-	-
				Isolated GIT	5	5	100.00	-	-	-	-	-	-
				Isolated pulmonary	2	2	100.00	-	-	-	-	-	-
				Total	18	16	88.89	-	-	2	11.11	-	-
**Overall**		**162**			162	92	56.79	17	10.49	29	17.90	24	14.80

### Prognosis of CMV EODs

Upon treatment, 40.5% (30/74) of the patients with CMV EOD/s with baseline CD4 count<30, responded to therapy, 12.2% (9/74) presented with static condition as they neither improved nor deteriorated in their CMV EOD/s status while 24.3% (18/74) of the subjects showed deteriorations during the study period.

In the CD4 range of 30–59cells/μl, the percentage of cases showing improvement in their CMV EOD/s status was 65.7 (46/70) and this increased to 88.9 (16/18) for subjects in the CD4 range of 60–100 cells/μl. 11.4% (8/70) of the subjects with CD4 range of 30–59cells/μl showed static condition while 12.9% (9/70) continued with deterioration in their CMV EOD/s status. 11.1% (2/18) of the subjects with CD4 in the range from 60–100 continued with deterioration. Amongst the total subjects with CMV EOD/s, 14.8% (24/162) died during the study period. Approximately 23.0% (17/74) of the subjects with CD4<30 and 10.0% (7/70) of the subjects having CD4 count between 30 and 59, died during the study period. Cases of mortality (17 out of the total 24 mortality cases) were highest amongst the study group with CD4<30. No death was recorded for the subjects with CMV EODs with CD4 between 60 and 100 (Table 2). Our results corroborated with some prior observation.[21]

### CMV EOD/s and Mortality

**Fig 1:**
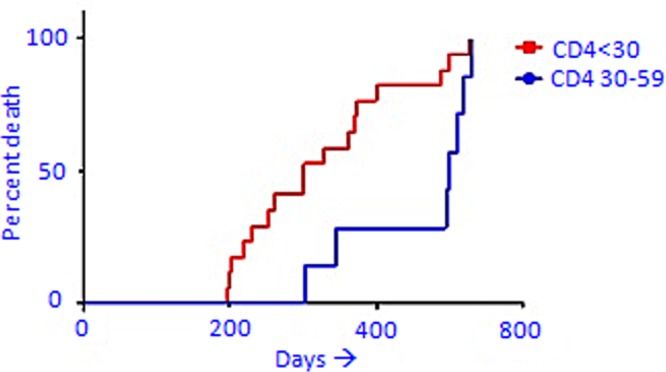
Comparison of death rates between subjects with CMV end organ diseases (EODs) with CD4 counts <30 and 30–59 (following the assumptions of the Cox proportional hazard model and after adjustments for age and gender).

Subjects with CD4 count between 30–59 who died during the study period had a median survival of 500 days after their entry to the study while those with CD4 < 30 had a significantly low median survival of 301 days after their entry into the study (Log-rank {Mantel-Cox} test: Chi square = 4.931,P = 0.03; Gehan-Breslow-Wilcoxon test: Chi square = 5.438,P = 0.02).The Hazard (Mantel-Haenszel) ratio was 2.641 (Fig. 1).

The baseline mean value of log 10 HIV-1 serum viral loads of the subjects who died (5.1) was significantly higher from those who survived the study period (4.5) (P<0.0001).The baseline mean value of log 10 CMV serum viral load of the subjects who died was 6.9 and those who survived the study period was 5.1 (P<0.0001).

### Determination of serum CMV viral load cut off value for development of CMV EOD/s

**Fig 2:**
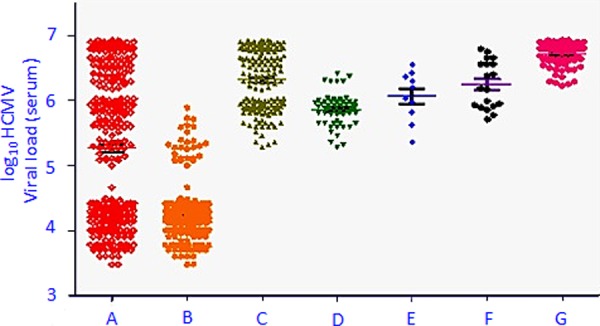
Serum CMV viral load amongst the subjects with varying manifestations. A: All CMV positive subjects (n = 337). B: CMV positive subjects with no CMV associated end-organ diseases (n = 175). C: Subjects with HCMV EODs (n = 162). D: Subjects with HCMV retinitis (n = 58). E: Subjects with HCMV pulmonary diseases (n = 10). F: Subjects with HCMV GIT diseases (n = 19). G: Subjects with disseminated HCMV diseases (n = 75).

The distributions of the CMV viral loads associated with different manifestations are described in Fig. 2 and Table 3. ROC curve was used for determining the cut off value of the serum CMV viral load for the development of EOD and EODs (disseminated CMV EOD). The CMV viral load cut-off value (expressed in log10 CMV viral load) for the development of CMV EOD was obtained as 5.4 (P<0.0001) and that for the development of disseminated CMV EODs was obtained as 6.4 (P<0.0001).

**Table 3:** Distribution of serum CMV* viral load amongst the subjects with varying manifestations.

Group ID	Category	N†	log_10_ CMV* DNA/ml serum	
			Mean (range)	Inter-quartile mean#
**A**	All CMV positive subjects	337	5.3 (3.5–6.9)	5.2
**B**	CMV positive subjects with no CMV associated end-organ diseases	175	4.3 (3.5–5.7)	4.2
**C**	Subjects with associated end-organ diseases	162	6.3 (5.5–6.9)	6.4
**D**	Subjects with CMV retinitis	58	5.9 (5.5–6.4)	5.9
**E**	Subjects with CMV pulmonary diseases	10	6.1 (5.3–6.6)	6.1
**F**	Subjects with CMV associated diseases of gastro-intestinal tract	19	6.2 (5.7–6.8)	6.2
**G**	Subjects with disseminated CMV diseases	75	6.7 (6.2–6.9)	6.8

* CMV: Cytomegalovirus

† N: No. of subjects in each group

#Inter-quartile means referred to the means of the mid-50% (between 25th & 75th percentiles) of the distribution mentioned to understand the distribution of the mean values excluding the potential outliers/less occurring values.

The obtained values were associated with a high sensitivity (97.5%; 95% CI-93.80% to 99.32% and 90.7; 95% CI—81.7% to 96.2% respectively) and specificity (96.0%; 95% CI-

**Fig 3:**
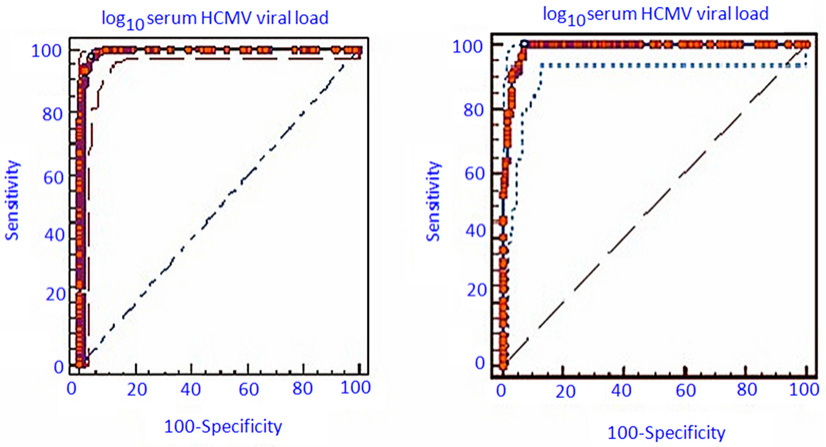
ROC curve for determining the cut-off value of serum CMV viral load for the development of (i) CMV end organ diseases (EODs) (left) and (ii) Disseminated CMV EODs (right).

91.9% to 98.4% and 97.0% 95 CI%- 94.1% to 98.7%) along with high positive predictive values (95.8%; CI- 91.5% to 98.3% and 89.5%; 95% CI 80.31% to 95.34%) and high negative predictive values (97.7%; 95% CI- 94.2% to 99.4% and 97.3%; 95% CI- 94.6% to 98.9%) respectively (Fig. 3 and Table 4).

**Table 4:** Sensitivity, specificity, positive and negative predictive values for the CMV viral load cutoffs associated with development and dissemination of CMV associated EODs.

Parameters	Values	95% confidence intervals	Values	95% confidence intervals
Viral load cut-off (log10 CMV* DNA/ml serum)	5.4	Not applicable	6.4	Not applicable
Sensitivity (%)	97.53	93.80–99.32	90.67	81.71–96.16
Specificity (%)	96.00	91.93–98.38	96.95	94.07–98.67
Positive predictive value %)	95.76	91.45–98.28	89.47	80.31–95.34
Negative predictive value %)	97.67	94.15–99.36	97.32	94.55–98.92
p value	<0.0001	Not applicable	<0.0001	Not applicable

### Association between CD4+ cell count and prognosis of the CMV EODs upon treatment

The mortality rate among the subjects with CMV EODs was significantly higher at a low CD4 count of <30 in comparison to those at a higher CD4 count of 30–59 [OR: 0.37 (95%CI 0.14–0.96) P = 0.04; RR:0.67 (95% CI 0.49–0.93) P = 0.01] and CD4 from 60–100 [OR:0.09 (95%CI 0.005–1.54) P = 0.09; RR:0.78 (95% CI 0.58–0.90) P = 0.001]. Similarly, significantly higher number of subjects responded to the therapy at higher CD4 count of 60–100 [OR: 11.11, (95% CI 2.5–50.00), P = 0.002; RR:1.48 (95% CI 1.18–1.82) P<0.001] and those having CD4 count from 30–59 [OR: 2.81 (95%CI 1.4–5.5) P = 0.003; RR: 1.64 (95%CI 1.18–2.27) P = 0.03].(Table 5)

**Table 5:** Association between the prognosis of CMV end organ diseases (EODs) with CD4 count among participating HIV positive patients (N = 400).

**CD4 range**	**Responded to therapy (%)**				**Static condition (%)**				**Deterioration (%)**				**Death (%)**			
	OR (95%CI)	P value	RR (95%CI)	P value	OR (95%CI)	P value	RR (95%CI)	P value	OR (95%CI)	P value	RR (95%CI)	P value	OR(95%CI)	P value	RR (95%CI)	P value
**<30**	Reference category				Reference category				Reference category				Reference category			
**30–59**	2.81(1.40–5.50)	0.003*	1.64(1.18–2.27)	0.03*	0.93(0.33–2.56)	0.14	0.97(0.60–1.56)	0.89	0.46 (0.19–1.10)	0.08	7.14(0.52–1.00)	0.047*	0.37(0.14–0.96)	0.04*	0.67(0.49–0.93)	0.01*
**60–100**	11.11(2.50–50.0)	0.002*	1.48(1.18–1.82)	<0.001*	-	-	-	-	0.39(0.08–1.85)	0.23	0.86(0.71–1.04)	0.13	-	-	-	-

* Results for which p value was<0.05

## Discussion

In patients suffering from HIV/AIDS, CMV reactivation and replication is accelerated due to compromised immune function particularly cell-mediated immunity. Cytomegalovirus infection in this patient group may result in EOD/s and is the leading cause of mortality [22,23].

Among the study subjects to have developed CMV EOD/s, retinitis was the commonest. 82.1% (133/162) of these subjects developed CMV retinitis (either isolated retinitis cases—35.8% or associated with other EODs-46.3%). Retinitis was a tell tale component of virtually every case except a few cases of isolated CMV induced GIT and Pulmonary diseases. So we suggest ophthalmoscopic examination and rigorous eye check up must be a compulsory practice in case of every HIV/AIDS patients having CD4 count ≤100 and / if the subject is having serum CMV viremia. This must be practiced even if the patient does not have any vision-related complains.

At lower CD4 range, CMV infection leading to disseminated CMV EODs was more common and the prognosis of CMV EOD/s was poor with higher percentages of mortality and cases showing deterioration in their CMV EOD/s status. There might be several possible explanations. First, poor prognosis could well be associated with high CMV and HIV-1 viral loads as well as with lower CD4 counts (<60) where both cell mediated and humoral immunity were virtually nonexistent. Second, subjects who have died or were not responding to therapy might have developed defects in their CMV specific immune responses or to other different opportunistic pathogens due to AIDS—associated immune-suppression beyond those typically experienced by patients with advanced AIDS but with comparatively higher CD4 counts. Such defects in immune response permit cell-to-cell CMV transmission leading to tissue necrosis associated with nonspecific inflammation and dissemination CMV infections leading to development of life threatening diseases [24]. Third, non-compliance to HAART and viral resistance to one or more components of HAART may be considered as a significant cause. Fourth, late diagnosis, delayed access to medical and health care and lack of awareness to potentially life threatening end organ dysfunctions posed by CMV might have played extensive roles for the ineffectiveness of therapy in these cases.

We also report a significantly higher (P<0.0001) CMV viral load in the serum of the HIV/AIDS subjects who developed CMV EOD/s with respect to those who did not develop CMV EOD/s. The mean of serum CMV viral load of the subjects diagnosed to have developed CMV EOD/s was 6.3 (range 5.3–6.9) and those who did not develop any CMV induced EOD/s was 4.3 (range 3.5–5.7) (Fig. 2).

In this study, we have determined the cut-off values of serum CMV viral load associated with the development of CMV induced EOD and disseminated EODs amongst the HIV/AIDS subjects using the ROC curve. The cut-off value thus determined has a very high negative predictive value for both the conditions indicating that large majority of the subjects with serum CMV viral load below this cut-off value will not develop any CMV associated EOD/s. Cut-off values for both CMV EODs and disseminated CMV EODs had high sensitivity and specificity (higher than 90%) with respective P values <0.0001 in both cases. Hence the areas under the ROC curves for both groups of patients indicated that the laboratory tests (serum CMV viral load) had the ability to distinguish between patients (Group I) with or without CMV EODs as well as the patents (Group II) with or without disseminated CMV EODs. The high positive predictive value is also indicative of the fact that proper interventions must be used so that the serum CMV viral load does not cross the cut-off value as a there is a possibility that large majority of these subject will then develop CMV associated EOD/s.

One important drawback of our study is that, since the subjects included in the study were late diagnosed HAART naïve HIV/AIDS patients with some of them developing early stages of CMV EOD/s, thus we cannot get the (almost) exact serum CMV viral load associated with the onset of CMV EOD/s. In a poor-resource setting collection of detailed data is not often optimally possible due to logistic issues including lack of electronic medical record management system. Hence lack of information on opportunistic infections, comorbidities, lack of optimal scope for interpretation of the serum CMV viral load cut-offs, lack of detailed information on socio-demographics other than age and gender, absence of a control HIV positive group (with no CMV EODs), lack of person-time data were the potential limitations in our study. While these issues were the potential limitations of our study still we tried our best to collect as accurate information as possible. A follow-up study at regular time interval is required whereby the (almost) exact CMV viral load associated with disease development could be determined upon onset of the symptoms of the disease. However, to minimize the error due to the forced approximation that had to be taken in determining the cut-off value we took two steps: first, we included in large cohort of study subjects—thus, by including a large pool of subjects with varied manifestations, we could minimize the unavoidable errors and thereby increase the precision of measurement of the parameter- second, we deliberately did not include in our study the subjects who were diagnosed with advanced CMV disease manifestations rather, only those subjects who were diagnosed to have developed only early stages of CMV disease/s were included.

The main strength of our study lies in the fact; that we are the first to establish a cut-off value of the serum CMV viral load associated with the development of CMV EOD/s amongst a large cohort of HIV/AIDS subjects. Our study firmly establishes serum CMV viral load to be a very important dependable parameter for the early prediction of CMV EOD/s development. This study can act as baseline information, which may play important role in determining future treatment strategies.

In summary, from this study we wanted to portray a clearer picture of the different clinical aspects of CMV infection leading to the development of CMV EOD/s amongst the HIV/AIDS patients. As expected, prognosis of CMV disease was poor in subjects with low CD4 count and was associated with higher death rates. We thus suggest proper steps to be taken before the CMV viral load crosses the cut-off value to avoid CMV associated EOD/s development among these HIV/AIDS subjects.
